# Complete mitochondrial genome of *Cyllorhynchites ursulus* (Coleoptera: Rhynchitidae)

**DOI:** 10.1080/23802359.2018.1483770

**Published:** 2018-07-12

**Authors:** Yung Kun Kim, Eunok Lee

**Affiliations:** Division of Ecosystem Services and Research Planning, Bureau of Ecological Research, National Institute of Ecology, Seocheon-gun, Chungcheongnam-do, Korea

**Keywords:** *Cyllorhynchites ursulus*, mitochondrial genome, Coleoptera

## Abstract

The complete mitochondrial genome of *Cylloryhunchites ursulus* was sequenced for the first time from its whole body using the next-generation sequencing method. This mitogenome was 14,508 base pairs in length, containing 13 protein-coding genes, 20 transfer RNA genes, and two ribosomal RNA genes. Its overall A, C, G, and T contents were 37.8, 17.8, 11.1, and 33.4%, respectively. Its A + T content (71.2%) was higher than G + C content. The phylogenetic result showed that although *C. ursulus* is a species of family Rhynchitidae, its phylogenetic position is closely related to family Attlabidae.

*Cyllorhynchites ursulus* belongs to order Coleoptera, family Rhynchitidae. Genus *Cyllorhynchites* is composed of four subgenera; subgenus *Cyllorhynchites* (five species from central and eastern Asia), subgenus *Hypocyllorhynus* (3 species from southeastern Asia), subgenus *Hyporhynchites* (five species from southeastern Asia), and subgenus *Pseudocyllorhynus* (nine species from eastern Asia) (Legalov 2007). Among them, *C. ursulus* is the only species belonged to genus *Cyllorhynchites* in Korea. In Korea, *C. ursulus* is not well-known species, and rather known as a pest insect because of their behavioral characteristics, which cut off the branches of acorn trees, drill a hole on acorn and lay their egg in acorn (Byeon 1991).

In this study, living specimens of *C. ursulus* were collected near the Namjangsa temple (N36°42′, E128°11′, elevation in 189 m) located on Noum-san mountain (Sang-ju city, Gyeongsangbuk-do, Korea) and stored in our laboratory at –80°C. We homogenized the whole body with pestle and extracted genomic DNA using Qiagen DNeasy Blood and Tissue kit (Qiagen Korea Ltd, Seoul, South Korea) following the manufacturer’s instruction. The complete mitogenome was sequenced on a Hiseq2000 platform using next-generation sequencing (NGS) technique (Illumina, San Diego, CA). Geneious 10.2.3 (Biomatters Ltd, Auckland, New Zealand), tRNA Scan-SE 1.21 (http://lowelab.ucsc.edu/tRNA Scan-SE/), and MITOS WebServer (http://mitos.bioinf.uni-leipzig.de) were used to assemble and annotate the mitochondrial DNA sequences. Phylogenetic tree that shows the comprehensive evolutionary relationship among other species was constructed on MEGA 5.05 based on maximum likelihood analysis with Tamura-Nei model (Figure 1) (Tamura et al. 2011).

**Figure 1.**
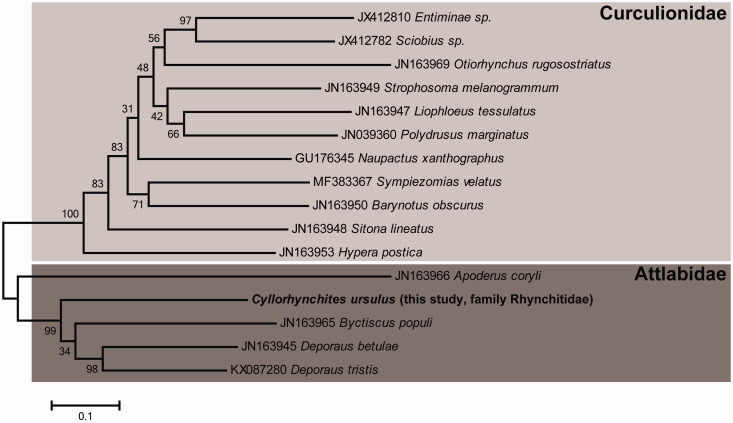
Maximum-likelihood phylogenetic tree of *C. ursulus* with other related species based on 13 protein coding genes (PCGs). The sequences accession number of the species used in phylogenetic analysis is shown in the figure.

The complete mitochondrial genome (GenBank Accession No. MH156809) was 14,508 base pairs in length, and includes 13 protein coding genes, 20 tRNA genes (ranging from 62 base pairs in tRNA-Arg to 70 base pairs in tRNA-Lys), and two rRNA genes. Its overall base contents are 37.8% for A, 17.8% for C, 11.0% for G, and 33.4% for T, which means AT content bias (71.2%). All 13 PCGs initiated with ATN(ATA, ATC, ATG, and ATT) codon, except for cytochrome c oxidase subunit II (COX2) started with TTG codon. Among 12 genes, three PCGs (ATP6, COX3, and ND4) start with ATG codon, three PCGs (ND2, ND3, and ND6) start with ATA codon, three PCGs (COX1, ATP8, and CYTB) start with ATC codon and three PCGs (ND4L, ND1 and ND5) start with ATT codon. Four PCGs (ND1, ND2, ND4, and COX2) terminate with T–, the incomplete stop codon. Six PCGs (COX1, COX3, ND4L, ND5, ND6, and ATP6) end with TAA, and three PCGs (ND3, ATP8, and CYTB) terminate with TAG codon.

The phylogenetic tree among the 16 species of Order Coleoptera (11 species of Family Curculionidae and five species of Attlabidae including *C. ursulus*) based on 13 PCGs shows that *C. ursulus* (family Rhynchitidae) is closely related with other species of family Attlabidae (Figure 1). In conclusion, the complete mitogenome of this species are essential data to understand phylogenetic position within order Coleoptera.
